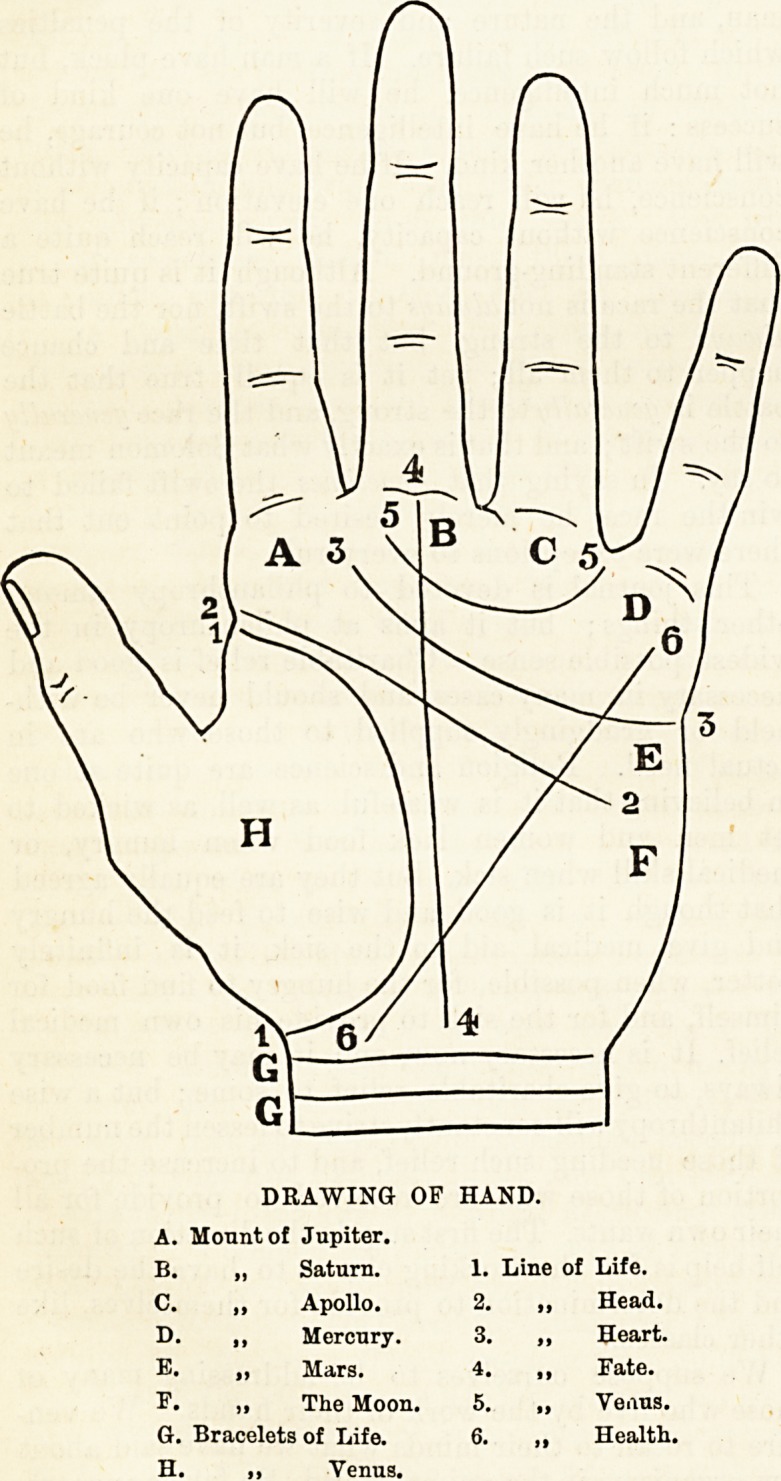# Palmistry Notes

**Published:** 1888-03-31

**Authors:** 


### Amusements for Convalescents.

### PALMISTRY NOTES.

By a Lady.

(*Concluded from page* 386.)

DRAWING OF HAND.

A. Mount of Jupiter.

B. „ Saturn.

C. „ Apollo.

D. „ Mercury.

E. „ Mars.

F. „ The Moon.

G. Bracelets of Life.

H. „ Venus.

1. Line of Life.

2. „ Head.

3. „ Heart.

4. „ Fate.

5. „ Venus.

6. „ Health.

For the indications of Saturn's Line (4) to be good, it should rise clear and straight, without breaks or crosses to mar its upward progress. From this we augur long life and a good disposition, and if the phalange of will—the first—on the thumb be long, we shall find a strength of will and individuality which will enable us to combat the difficulties we are sure to encounter on our way through life. If the Line is twisted, it may be surmised that the possession of an uncertain and irritable temper has marred our success during the period whence this twisting is observable, and should the line be *split* also, this uncertainty of temper is the result of ill-health. If throughout its course the line present an uneven appearance and the palm is much rayed, an over-anxious and too sensitive temperament is to be feared; the evil effects of this development will be much mitigated if the hand is *hard*, showing *resisting* power, but with a *soft* palm, this over nervous activity will render its possessor discontented and prone to brood over matters that are more imaginary than real. *Breaks in the Line* always indicate troubles; broken in the centre, mental distress, caused by moral struggles; at the Heart Line (3) grief caused by bereavement or disappointment; at the Head (2) illness which may affect the brain. Domestic unhappiness is indicated by a line from Venus (*h*) cutting the Line of Saturn. Troubles in childhood by a broken and frayed condition of the line at its outset; if a star is found upon Venus (*h*) it has been accompanied by loss of money and position owing to the death of a parent. *Crosses* indicate changes which, if the Life and Head Lines are similarly marked, have arisen from the loss of friends or from some unfortunate venture or miscalculation on our own part. A star at the termination of the Line upon the mount (*b*) tells of trouble arising late in life from the wrong-doing of others. An inclination to travel is shown by a forking of the line at the commencement, one ray going towards Venus (*h*) and one to the Moon (*f*). If this is found in the left hand only, journeys have been *contemplated*, but if the birfurcation occurs in *both* hands, with travel lines from the wrist on to the mount of the Moon, the journeys will be undertaken. Another signification is attached to a forking of the Line, which sends a ray towards the centre of the hand and another to Venus. It is said to indicate a fatal influence over one of the opposite sex, and in proportion as our hand is a good or bad one, so will that influence be exercised. *Absence of the Line* is indicative of a commonplace, negative existence, and in the character of the person whose hands display this defect, there will be a corresponding absence of light and shade.

We now come to a Line, which though not appearing on the drawing before us, is too important to be omitted from this brief description of the Lines in the Palm. It is called the *Line of Apollo* (*c*), and for its indications to be happy, it should trace a fine clear furrow on the mount under the third fingers We then foretell for its possessor distinction in art or literature, with wealth inherited or acquired by ability and industry; good fortune also is augured from the influence and good offices of someone in power who is ready and willing to befriend us. From the strength or weakness of the line we estimate whether our efforts towards distinction have been successful or otherwise. We have seen in this line, as in others, that the *clearer* and *firmer* the line is, like the character and career which it is supposed to reflect, the more certain are the fortunate and happy premises to be gleaned from it. Therefore, when the line is broken up instead of being firm and strong, which is indicative of wealth of celebrity arising from the cultivation and exercise of artistic gifts and inspiration, we judge that vacillation, caprice, and want of continuity have diverted our artisti ability from the one direction in which by steady perseverance we might have achieved distinction, into a fatal frittering of talents which has resulted in neither artistic nor monetary success. When the line is good, a serious barrier to complete success is found by a ray from Saturn (*b*) cutting Apollo's Line, which indicates want of means to prosecute the cultivation of our artistic gifts. A good development of the mound (*c*) with Saturn's Line (4) rising from the Moon (*f*) promises the influence of a rich and powerful person, which goes far to amend the effect of this thwarting ray. Three upright lines on the mount promise gratified ambition from the exercise of literary and artistic ability. An unfailing indication of wealth is the presence of a well-formed line with Mercury (*a*) and Jupiter (*a*) prominent, giving celebrity from scientific ability and high moral character. Too long a Line of the Head, twisted fingers, or a very long line of Apollo, with a hollow palm, are significant of the higher attributes of the line being degraded to exercise of selfish and ignoble ends, such as the attainment of wealth or ambition for mere personal and material gratification. Wealth and celebrity from the proper exercise of artistic inspiration is shown by the line tending towards the Line of Saturn or Fate (4).

*The Line or Circle of Venus* (5) more rarely met with than the other lines of Life, Head, Heart, etc., gives, in a *good hand*, energy and force to a character which will expend itself in a desire to give pleasure to others, while not without a natural desire for personal appreciation also. In any pursuit or occupation in which its owner may engage the presence of the line, particularly if it end *low* on Mercury (*d*) points to enthusiasm and vigour in the prosecution of it. An inherent love of gaiety and amusement accompanies the line also. It's evil indications in *a bad hand* are indolence, vanity, and caprice; in short, want of moral stamina, and the pursuit of pleasure at all risks for mere selfish gratification, is a marked characteristic of the line when the hand is a bad one. In a *weak* hand (and I hope by this time my readers are able from the foregoing remarks on the different types of hands, with their consistency and line markings, etc., to judge as to the comparative strength or weakness of a hand, and its more or less pronounced tendencies towards good or evil, as evinced by certain signs and developments) in a *weak hand* with the mounds (*h*) and *(f)* developed the excess of feeling, of *sentiment* rather, associated with the Line will, particularly if the palm is covered with innumerable small Lines, develop caprice and restlessness, with a constant desire for excitement. There is always a certain amount of literary and poetic power with this Line, whether the perseverance necessary for its successful exercise is present or not.

*The Line of Health*, called also the Liver and the Hepatic Line starts from the foot of the Line of Life (1) and runs from thence in a diagonal direction to the Mount of Mars (*e*) or to Mercury (*d*). A clearly marked line promises good health, aided by that most important function—a good digestion. When the Line ends on Mercury (*d*) it augurs long life, in spite of weak health, or serious illness, shown by a broken or uneven state of the Line; ending thus, and forked at the extremity, ambition and a capacity for command with high imaginative and intuitive gifts will be present. The presence of a second line running *parallel* with the Health Line enforces these indications. Absence of the Line is said to indicate a vivacious and active temperament. A glance at the *Lines on the Wrist* (*g*) will bring us to the end of these "Notes on Palmistry." The more clearly these lines, usually three in number, are marked the greater the probability of our life being long and happy. Indistinct or uneven, carelessness in money matters is indicated, while vanity and evasion are shown by a severing of the Lines under Saturn's Line (4). If chained, a life of honourable labour, crowned with ease and affluence is to be looked for. A star or cross found on the wrist foretells inheritance late in life. Lines from the wrist vary in their signification according to the mount on which they end; on *the Moon (f)* they mean journeys which will be short or long in proportion to the length of the lines. A line from the Rascette (*g*) crossing the hand to Jupiter (*a*) indicates an unusually long and lucky journey. A line to Apollo literary or artistic success, or marriage with some one endowed with these gifts; to Mercury (*d*) success in business or scientific pursuits, or wealth from an unexpected source. We have now traversed the map of the hand, and I have endeavoured to show in the short articles which preceded those on the *Lines of the Palm*, how by the study of the *formation of the hand* we are able to judge, from the type to which it belongs, of the pursuits or occupation to which we shall naturally incline, and whether the tendencies of our nature are of a *practical* or *artistic* order. From the *Lines of the Palm* we have learned that by a proper exercise of *will*, aided by that highest form of courage—*moral* courage, directed and controlled by the Divine gift of reason, yielding to the guidance of a Higher power, we may achieve a victory over self which would otherwise be impossible. That lines are placed in our hands for some good purpose I do not doubt. With us must rest the responsibility of heeding or neglecting their signals; the power to mitigate the effects of an evil or significant sign is given to everyone in greater or lesser degree, lines are known to change and alter from time to time, old lines fade and new ones appear. We all know how the steady persistence in a weak or evil course of action reduces a character to a condition, distinguished by the term *Fatal*, so that the power to form and direct our *fate* or so-called *destiny*, lies more literally in our own hands than many of us, from indolence or disinclination to alter our mode of life, may care to believe. We cannot all be brilliant, but we *can* be honest, and energetic, and true, for we are all gifted in one direction or another, and within ourselves *lies* the power to utilize the mental and moral attributes with which we are endowed. Let us look to our hands then, believing that we hold our Fate *there*—there for good or evil, more truly than we have hitherto realized.

## Figures and Tables

**Figure f1:**